# Investigation of Potential Reservoirs of Non-Tuberculous Mycobacteria in a European Sea Bass (*Dicentrarchus labrax*) Farm

**DOI:** 10.3390/pathogens10081014

**Published:** 2021-08-11

**Authors:** Davide Mugetti, Katia Varello, Paolo Pastorino, Mattia Tomasoni, Vasco Menconi, Elena Bozzetta, Alessandro Dondo, Marino Prearo

**Affiliations:** Istituto Zooprofilattico Sperimentale del Piemonte, Liguria e Valle d’Aosta, 10154 Torino, Italy; katia.varello@izsto.it (K.V.); paolo.pastorino@izsto.it (P.P.); mattia.tomasoni@izsto.it (M.T.); vasco.menconi@izsto.it (V.M.); elena.bozzetta@izsto.it (E.B.); alessandro.dondo@izsto.it (A.D.); marino.prearo@izsto.it (M.P.)

**Keywords:** fish farm, *Mycobacterium marinum*, mycobacterial infection, prevention, zoonoses, biofilm, hazard analysis

## Abstract

Fish mycobacteriosis is a widespread global problem caused by species of non-tuberculous mycobacteria (NTM). *Mycobacterium marinum* is one of the species most often involved in disease episodes of aquarium and farmed fish. Since there is currently no available effective therapy or vaccine, a prompt search for routes of entry is key to limiting the damage induced by the disease. Here we report a case of mycobacteriosis follow up in a European sea bass (*Dicentrarchus labrax*) farm located in Northern Italy, in which environmental samples and newly added fish batches were analyzed. Samples from fish present on the farm, sediment, and periphyton all resulted positive for *M. marinum*, whereas the new fish batches and the water samples resulted negative. The environmental resistance of NTM (alcohol-acid resistance, biofilm formation) and the lack of prophylactic and therapeutic strategies make these diseases difficult to manage. Prompt identification of biotic and abiotic reservoirs, combined with good zootechnical hygiene practices, are the most effective measures to control fish mycobacteriosis in intensive farms.

## 1. Introduction

Fish mycobacteriosis is a complex of infectious diseases caused by bacteria of the *Mycobacteriaceae* family [1]. Nearly 200 species of microorganisms in this family share unique characteristics [2]: non-motile, alcohol-acid resistant, aerobic pleomorphic bacilli with an environmentally resistant bacterial wall composed chiefly of mycolic acids. Due to the particular composition of the wall, Ziehl-Neelsen stain (ZN) is used for initial diagnosis [3].

The best-known species of the genus *Mycobacterium* (*Mycobacterium tuberculosis*, *M. leprae*) are human pathogens and do not cause fish mycobacteriosis; however, most other species cause disease in fish. These mycobacteria do not cause tuberculosis; they are generally identified as non-tuberculous mycobacteria (NTM) [4]: the best known NTM that cause disease in fish is *M. marinum* [5]. This photochromogenic bacterium produces smooth to rough yellow colonies on Löwenstein–Jensen medium after incubation for 7 or more days (slow-growing) [6]. *M. marinum* can infect fresh and salt-water fish species [7,8].

Prevalent in aquarium fishes, *M. marinum* infections are often also reported in farmed fish exposed to stressful conditions that favor disease onset [9,10,11]. Common clinical signs are ulcerative lesions of the skin and nodules of the parenchyma of internal organs (spleen, liver, kidney) [12]. The disease is often chronic and associated with drip mortality, though cumulative mortality can also be severe [1]. The zoonotic potential of *M. marinum* besides it being a fish pathogen [5] is noted in cases of skin disease (fish tank granulomas) in humans who handle fish or work in aquaculture [13,14,15,16,17]. *M. marinum* can also cause systemic infections in persons with immune deficiency or autoimmune disease [18]. 

Infection caused by *M. marinum* in a fish farm is highly problematic. As there are no specific external clinical signs, correct diagnosis of the disease is challenging because granulomas of internal organs can also be caused by other infectious and non-infectious agents [9]. No treatment is available, which is why fish mycobacteriosis needs “to be managed” by management and environmental factors [19]. Here we report a case of mycobacteriosis due to *M. marinum* in European sea bass (*Dicentrarchus labrax*). In the search for potential pathogen reservoirs, newly added fish and sediment, water, and periphyton samples were analyzed to detect the presence of NTM.

## 2. Results

### 2.1. Anatomopathological, Parasitological, and Bacteriological Analysis

**Figure 1:**
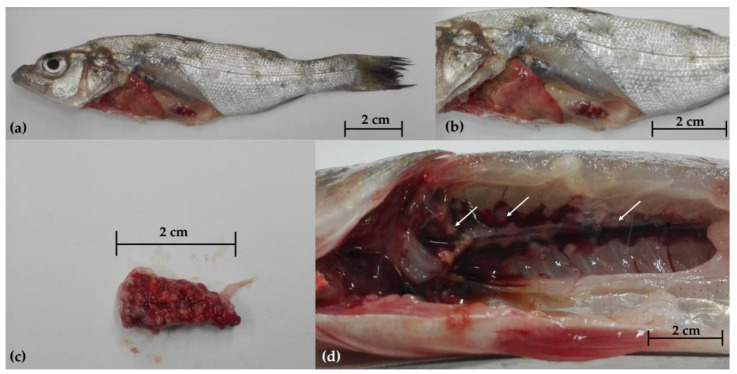
Gross pathology of European sea bass (*Dicentrarchus labrax*) during the first sampling; (**a**) external examination showing emaciation and skin lesions; miliary nodules in the spleen (**b**,**c**) and kidney (**d**).

During the first sampling, necropsy disclosed emaciation, skin erosions (Figure 1a), miliary nodules in the spleen (Figure 1b–c), liver, and kidney (Figure 1d). Parasitological and bacteriological analysis revealed no primary or opportunistic infections. During the second sampling, the newly added fish showed no lesions and tested negative for parasites and bacteria. 

### 2.2. Histopathological Examination

**Figure 2:**
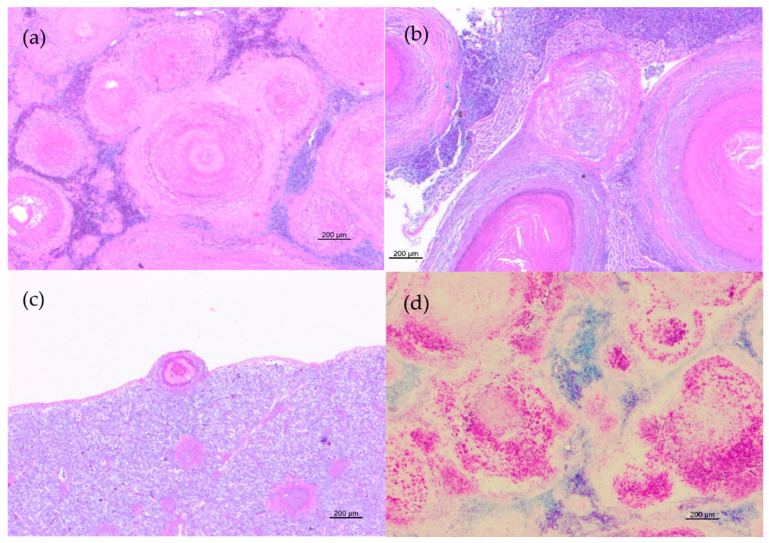
Histopathological features of granulomatous lesions in trout tissues. (**a**) Spleen: multiple coalescing necrotizing granulomas with destruction of normal organ architecture (HE); (**b**) Liver: different stages of granulomas. Subcapsular granuloma with a distinct necrotic core surrounded by epithelioid cells and spindle-shaped cells and multiple parenchymal granulomas characterized by centralized epithelioid cells with eosinophilic cellular debris in the center of the lesion (HE); (**c**) Kidney: multiple granulomas with a central eosinophilic area of necrosis surrounded by inflammatory cells and enclosed by a thin capsule (HE); (**d**) Spleen: numerous acid-fast bacilli in granulomas (ZN).

Histopathological examination disclosed lesions referable to mycobacterial infection in the spleen and the kidney of all animals and in the liver of four animals. More severe lesions were observed in the spleen and the kidney, where 70–80% of the normal tissues was replaced by multiple coalescing necrotizing granulomas (Figure 2a,b). One or rare multiple granulomas were found in the liver (Figure 2c).

Granulomatous lesions were characterized by epithelioid cells, foamy macrophages sometimes containing dark brown pigment in the cytoplasm, lymphocytes, and plasma cells with large eosinophilic necrotic areas partially or completely enclosed by a thin capsule. All lesions resulted positive at ZN staining and displayed moderate-to-large amounts of acid-fast bacilli (Figure 2d).

### 2.3. NTM Culture Screening

About 3 weeks after incubation, yellow colonies grew on Löwenstein–Jensen and Stonebrink medium from organ (spleen, liver, kidney) tissue samples from 17 fish collected during the first sampling (17/80, 21.3%). 

The same yellow colonies grew on the periphyton and sediment samples; however, no colonies grew following incubation for 2 months of the water samples or the tissues from the fish collected in the second sampling. Following Kinyoun staining, all colonies were classified as alcohol-acid resistant, rod-shaped bacteria. Table 1 presents the results of the bacteriological tests.

### 2.4. Biomolecular Analysis

**Figure 3:**
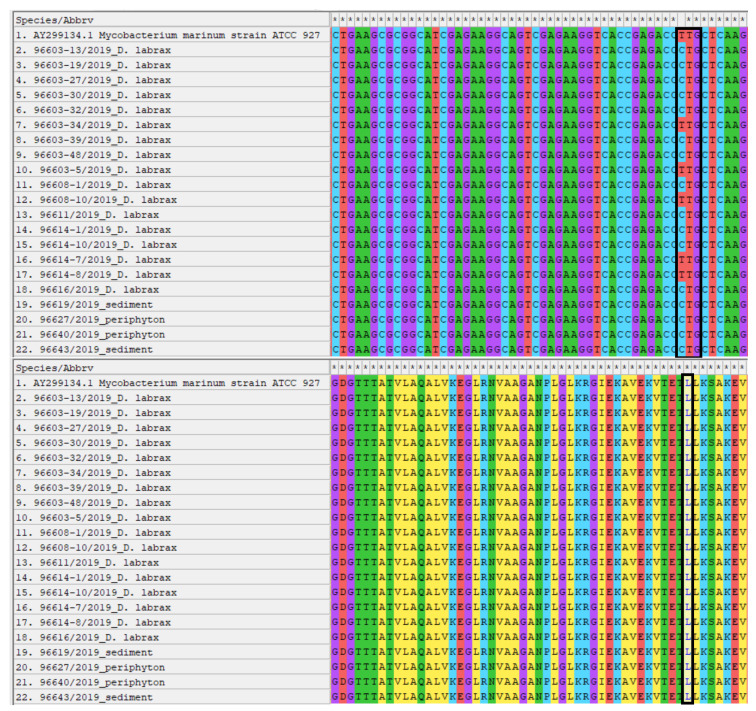
Alignment by MEGAX of the nucleotide and amino acid sequences of the isolated *M. marinum* strains. The sites of interest where differences were found between the strains are highlighted.

All colonies underwent PCR assays and yielded a band identical to the positive control (~440 bp) in electrophoresis, so they were classified as belonging to the genus *Mycobacterium*. Sequence analysis by BLASTn identified the colonies as *M. marinum*, with 100% identity. The sequences showed a single difference (C/T) in the nucleotide sequence (sequence position 234, corresponding to nucleotide position 852069 of the reference strain *M. marinum* ATCC 927 genome), resulting in a silent mutation (CTG/TTG both translated into leucine). The sequences were deposited in GenBank database with the following accession numbers: MZ733734, MZ733735. Differences in nucleotide sequences with respective amino acid sequences are shown in Figure 3.

## 3. Discussion

Fish mycobacteriosis is a troublesome problem for fish farms, especially for salt-water farms [8]. The most common bacterial species that causes these infectious diseases is *M. marinum*, although several related species have also been reported [20,21,22,23]. Because there is no therapeutic and prophylactic treatment for NTM infections [24], fish farms need to have appropriate management practices in place to deal with the disease. 

Following the owner’s report of drip mortality of European sea bass in multiple tanks, two waves of sampling were conducted on the farm. The initial suspicion was mycobacteriosis, given the ulcerative skin lesions and the constant, low mortality rate. Following this hypothesis, we decided to sample potentially infected fish and also to take environmental samples (tank water, sediment, periphyton) that could possibly act as a reservoir for the mycobacteria. These matrices are known sources of NTM [25,26,27] owing to their environmental resistance and potential for biofilm formation [28,29,30]. In a second sampling wave, new batches of fish were sampled because the farm always added fish from the same supplier. This second analysis was done to exclude the entry of pathogens (mycobacteria) via the new batches.

Anatomopathological examination disclosed nodules in the parenchymatous organs, primarily the spleen, followed by the kidney and the liver. These are common signs of mycobacterial infection in farmed fish, though other bacterial species (e.g., *Nocardia* sp., *Photobacterium damselae*) cannot be excluded [31,32]. Correct differential diagnosis relies on histological analysis to characterize the nodules and identify acid fast bacilli. Histological analysis confirmed our initial suspicion and enabled us to identify ZN-positive bacilli and characterize the granulomas. It is also possible, however, that some partially ZN positive bacteria (e.g., *Nocardia* sp.) may cause identical lesions [31]. The parasitological and bacteriological tests ruled out the presence of other primary or occasional pathogens. 

A specific culture test was performed to confirm the NTM presence. After three weeks of incubation, colonies grew from organ tissue samples (liver, spleen, kidney) from 17 fish (17/80, 21.3%). Biomolecular analysis initially identified the isolates as *Mycobacterium* spp. (genus-specific PCR) then, following Sanger sequencing, as strains of *M. marinum* with an identity percentage of 100%. The difference of a single nucleotide between the various isolates was found in the *hsp65* gene portion. This difference is phenotypically irrelevant, as the expressed proteins are identical on analysis of the resulting amino acid sequences (Figure 3). These results confirmed our initial hypothesis for this most common etiological agent of fish mycobacteriosis in marine fish. Infection with *M. marinum* have been reported in European sea bass [33] and in other farmed species such as gilthead sea bream (*Sparus aurata*) [34], mullets (*Liza ramada*) [35], and meagre (*Argyrosomus regius*) [36].

Having identified the etiological agent for which no antibiotic is available, we attempted to identify the potential reservoirs of *M. marinum* on the farm. For this purpose, environmental samples (tank water, sediment, periphyton) were analyzed, then the new fish before being added into the tanks. The sediment (2/2, 100%) and the periphyton (2/2, 100%) samples tested positive for *M. marinum,* whereas the water and the new European sea bass batches tested negative, confirming that monitoring the environment in which the fish are reared is crucial for disease control. 

The sediment on the bottom of tanks and the periphyton on the edges and walls constitute an ideal substrate for the persistence and replication of mycobacteria. Cases of mycobacteria isolation have been documented in controlled environments where running water is present (e.g., water supply systems) [37]. 

While analysis of the water samples was negative for *M. marinum*, this did not completely rule out its presence since the microbial load might not have been detectable by the method we used. Differently, the two other matrices (sediment and periphyton) can provide sites of NTM accumulation, samples from which positive culture were obtained. Finally, analysis of the newly added fish ruled out the possibility of mycobacteriosis originating from their addition to the tanks. Although mycobacterial infections are generally chronic and take time to develop, analysis of added batches is a step of fundamental importance in prevention. For example, cases of other pathologies (e.g., Acipenser Iridovirus European [AcIV-E]) showed that analysis was fundamental in preventing episodes of massive mortality on the farm after the addition of infected fish [38]. 

Here we report a well-known problem in sea bass farms: fish mycobacteriosis sustained by *M. marinum*. Although the data describing the lesions and isolation of the pathogen are not new, we wanted to extend current knowledge about them by analyzing different matrices that can provide a reservoir for NTM. This approach may be systematically applied in the prevention and management of NTM infection. Elimination of environmental sources of mycobacteria, combined with good zootechnical hygiene practices, are the only means currently available to reduce losses caused by NTM in fish farms. Treatment for NTM is challenging in humans and is not amenable to the containment of fish mycobacteriosis. The most promising means to manage these diseases is by vaccination. Although experimentally available, it is not applicable on a large scale [39]. The only means to remove mycobacteriosis on the farm is by eradicating it, followed disinfection of the physical structures, which results in high economic losses for farmers. The simplest and most effective approach is to follow Hazard Analysis and Critical Control Points (HACCP) rules in an effort to identify the risk factors and the measures to prevent and contain them.

## 4. Materials and Methods

### 4.1. Sampling

An inshore farm in northeastern Italy was sampled following a report of cases of drip mortality. The first sampling was carried out in November 2019, in which the infected tanks were sampled: European sea bass (80 individuals; length 12.3 ± 3.0 cm, weight 27 ± 4.8 g), water (two samples), sediment (two samples), and periphyton (two samples). The second sampling was conducted in February 2020, when new fish were added (64 individuals; length 10.4 ± 0.8 cm, weight 13.5 ± 3.3 g). The first sampling was carried out to determine the presence of mycobacteria in the fish and the environmental samples, while the second sampling was done to rule out pathogen entry due to the addition of infected fish (diseased fish and new ones from the same supplier) (Table 1).

### 4.2. Anatomopathological, Parasitological, and Cultural Analysis

The fish were euthanatized on the farm and sent refrigerated to the Fish Diseases Laboratory of the Istituto Zooprofilattico Sperimentale del Piemonte, Liguria e Valle d’Aosta (Turin) within 24 h, where necropsy, parasitological, and bacteriological tests were carried out. For the anatomopathological examination, the fish were macroscopically inspected by means of sterile tools and the lesions noted. Internal organs (liver, spleen, kidney) were removed for culture to detect NTM. 

The gill lamellae, the cutaneous, and the gill mucus were inspected for parasites using an optical microscope (BX40 Clinical Microscope; Olympus, Tokyo, Japan) at increasing magnification (10×, 20×, 40×) The coelomic cavity and the intestinal package were examined macroscopically and microscopically for endoparasites.

For bacteriological culture, brain and kidney samples were aseptically removed using 10-μL sterile loops, inoculated on Columbia blood agar (Liofilchem, Roseto degli Abruzzi (TE), Italy), marine agar, and thiosulfate citrate bile sucrose (TCBS) agar and incubated at 22 ± 2 °C for 72 h. Isolates were identified using matrix-assisted laser desorption ionization time-of-flight mass spectrometry (MALDI-TOF MS) on a VITEK MS system (bioMérieux S.A., Marcy-l’Étoile, France).

### 4.3. Histopathological Examination

The internal organs (spleen, liver, kidney) from 10 fish with nodules were prepared for histopathology. The samples were fixed in 10% neutral-buffered formalin and processed by standard paraffin wax techniques. They were cut in 4 ± 2-μm sections and stained with hematoxylin-eosin (HE) and ZN histochemical acid-fast stain. Sections were observed microscopically at increasing magnification (4×, 10×, 20×, 40×) on an Axio Scope.A1 microscope (Zeiss, Jena, Germany). Mycobacterial lesions were evaluated and classified as described by Gauthier et al. [40] and the ZN stain was considered positive in the presence of bright red staining rods. The pictures were captured using a Zeiss Axiocam 105 color camera at a resolution of 300 dpi. 

### 4.4. NTM Culture Screening

The internal organs were resuspended in physiological solution and homogenized using a Seward™ Stomacher™ Model 400 circulator lab blender (Thermo Fisher Scientific, Waltham, MA, USA). After decontamination with 1.5% cetylpyridinium chloride monohydrate (AppliChem, Darmstadt, Germany) solution for 30 min, the homogenates were centrifuged for 20 min at 3000 rpm. A pellet (10 μL) was inoculated using a sterile loop on Löwenstein–Jensen medium (Microbiol, Uta-Cagliari, Italy) and Stonebrink medium (Microbiol) tubes. Two tubes were used for each medium; one was incubated for 60 days at 28 ± 1 °C and the other at 37 ± 1 °C; the tubes were checked daily. The colonies were tested for ZN with cold-modified carbolfucsin (Kinyoun staining). Positive ZN colonies were identified using a genus-specific polymerase chain reaction (PCR) assay and Sanger sequencing.

For the environmental samples, 1 L of tank water was filtered following the protocol described in Le Dantec et al. [41]. The sediment was then processed as described for the tissues. The sediment samples were used directly, decontaminated, and centrifuged as described above. Finally, the periphyton samples were mechanically minced and then processed like the rest of the samples. 

### 4.5. DNA Extraction and PCR Assay

The isolates were suspended in 200 μL of water for molecular biology (Sigma-Aldrich, St. Louis, MO, USA). Bacterial DNA was extracted by heating to 96 °C for 10 min and then cooling to −20 °C. The extracts were stored at −20 °C following thawing and centrifugation to remove cell debris. For species identification, a fragment of ~440 bp of the 65 kDa heat shock protein (*hsp65*) [42] was amplified on a 2720 Thermal Cycler (Applied Biosystems, Waltham, MA, USA). PCR was performed in a volume of 50 μL containing 1X TaKaRa Premix Ex Taq version 2.0 (Takara Bio Inc., Shiga, Japan) and 20 μM of each primer. A reference strain of *M. marinum* (DSM 44344 / ATCC 927 / NCTC 2275) was used as PCR-positive control and ultrapure water as negative control. The PCR products were highlighted by gel electrophoresis on 2% agarose (Merck, Darmstadt, Germany), prepared using tris acetate-EDTA (ethylenediaminetetra-acetic acid) buffer 1X (Merck) and GelRed^®^ nucleic acid stain (Biotium, Fremont, CA, USA); the electrophoretic race was carried out for 50 min at 100V and the molecular size was determined by AmpliSize molecular ruler 50–2000 bp ladder (Bio-Rad, Segrate, Italy). Amplicons were purified using Extractme DNA Gel-Out kit (Blirt S.A., Gdansk, Poland) and sequenced by Sanger sequencing with primers Tb11 and Tb12 at a concentration of 100 μM. Forward and reverse sequences were assembled using ClustalW [43]; the consensus was matched with the NCBI database using Nucleotide BLAST to determine isolate species. The sequences were analyzed by MEGAX to detect differences in genomic and amino acid sequences [44]. 

## Figures and Tables

**Table 1:** Samples tested for mycobacteria.

Sample	Number of Samples	Positive	Negative
European sea bass (1st sampling)	80	17	63
European sea bass (2nd sampling)	64	-	64
Sediment	2 ^(a)^	2	-
Periphyton	2 ^(a)^	2	-
Water	2 ^(b)^	-	2

^(a)^ Each sample consisted of an aliquot taken from a 15-mL Falcon tube; ^(b)^ each sample consisted of a 1-L aliquot contained in a sterile plastic bottle.

## Data Availability

Not applicable.
